# Non-Pharmacological Management of Charcot–Marie–Tooth Disease: A Case Report

**DOI:** 10.3390/muscles5030059

**Published:** 2026-08-25

**Authors:** Irene Carantini, Roberto Cannataro, Francesco Ferraro, Erika Cione

**Affiliations:** 1 Physical Medicine and Rehabilitation Unit, Department of Neurosciences, ASST Carlo Poma, 46100 Mantova, Italy; irene.carantini@gmail.com (I.C.); francesco.ferraro@asst-mantova.it (F.F.); 2 ACMT-Rete per la Malattia di Charcot-Marie-Tooth OdV Association, 00167 Rome, Italy; 3 Department of Biology, Ecology and Earth Sciences, University of Calabria, 87036 Rende, Italy; 4 Research Group in Sports Nutrition (DBSS-Nut), Dynamical Business & Science Society—DBSS International SAS, Bogotá 110311, Colombia; 5 Department of Pharmacy, Health and Nutritional Sciences, University of Calabria, 87036 Rende, Italy; erika.cione@unical.it; 6 Galascreen Laboratories, University of Calabria, 87036 Rende, Italy

**Keywords:** CMT, strength training, inflammation, supercompensation

## Abstract

Charcot–Marie–Tooth (CMT) is a rare, genetic, slowly progressive disorder that affects nerve conduction, particularly in the limbs and, therefore, the muscles. Phenotypes vary, but the impact on quality of life is always present. CMT1A is the most prevalent type. There is no pharmacological cure, so physiotherapy is essential, but nutritional and exercise aspects are rarely considered. In this case report, we demonstrate how, even in this condition, effective results can be achieved with strength training if properly supervised, coordinated with physiotherapy, and combined with an appropriate nutritional plan.

## 1. Introduction

Charcot–Marie–Tooth (CMT) is a rare degenerative disease and one of the most common hereditary peripheral polyneuropathies with a prevalence of around 1:2500 [1]. CMT phenotypes are heterogeneous due to alterations in more than 100 genes. Two forms, demyelinating and axonal [2], cause a reduction in nerve conduction or nerve action potential amplitude. The onset of CMT occurs in the first two decades of life, and symptoms slowly progress from the distal to the proximal parts of the limbs, leading to motor and sensory impairments and, consequently, impairing patients’ quality of life [3]. CMT1A is the most frequent form of the disease; due to duplication of the short arm of chromosome 17, which hosts the PMP22 gene [4], it presents with progressive symptoms in childhood and adolescence. It causes hypoesthesia and hypotonia of the feet and leg muscles, altering agonist-antagonist balance and resulting in musculoskeletal deformities such as pes cavus, foot drop, hammer toes, tendon contractures, and subsequent difficulties with walking (stepping gait) and postural stability. It can also involve the upper limbs in a length-dependent progression, resulting in weakness of intrinsic hand muscles and difficulties with grasping and handling; psychological consequences are frequent [5,6].

Moreover, a recent study demonstrates that disability is more severe in underweight or obese children with CMT and becomes greater year by year according to worsening BMI [7], with a possible correlation to muscle mass; this is because the impairment of CMT, especially in the most widespread type 1A, impairs motor neuron functions and therefore muscle mass and strength. Therefore, continuous stimulation and muscle tropism can slow the condition’s progression. To date, there are no effective drugs available for patients with CMT, and the treatment of the disease relies primarily on physiotherapy and surgery [8]. Strength training (ST) is now used to support various pathological conditions; our group has used it, for example, to support cases of osteoarthritis [9], but in CMT, it seems from preliminary studies that it could be a safe and effective practice, indirectly affecting mood and behavior [10,11]. ST can maintain and strengthen proximal and distal limb muscles and improve performance during activities of daily living in CMT [10,12,13,14,15], and the right balance between compliance, progression, safety and effectiveness of training is crucial for these people; from Conde’s report [13] it is clear that there is still no well-defined protocol, especially in adults; on the other hand it should be intuitive that in the loss of neuronal function also muscular functionality and strength are affected; therefore resistance training should be strongly recommended; certainly with adequate attention to the execution of the exercises and the progression of the load; in the work of Ramdharry et al. [14] improvements in terms of strength are reported but this does not translate into an improvement in gait speed; the main limitations of both reports is that most of the programs were carried out without direct supervision, i.e., a program was provided to be carried out independently; in our case we supervised every single training session, for this reason we decided to bring this research to the attention of the scientific community. For a training program to be effective, at least two other aspects must be considered: rest and refueling [16]; regarding the first, the indications can be generic, but they mainly concern the periodization of training; of fundamental importance is nutrition and possibly integration; certainly a caloric intake greater than the calories consumed must be guaranteed to favor an environment aimed at anabolism; protein sources of high biological value should provide part of these excess calories, but, especially if the subjects are young, an adequate intake of vitamins, minerals, fibers, and polyphenols must be guaranteed; supplements such as omega3 could be very useful in managing recovery from physical activity but also for a general health objective [17]; at the moment there is no specific literature on CMT, so what we did was to figure out following the idea of managing inflammation and grant an amount of nutrients that allow a productive effect of exercise.

## 2. Case Description

The subject began receiving care at our facility in 2019. He is an 18-year-old male, 181 cm, and 63.5 kg; he started to feel symptoms in 2009 (frequent falls and some difficulties in coordination and sports); in his family, the father is affected. He read and approved the publication of the manuscript by written informed consent. He was genetically diagnosed with CMT1A in 2012; previously, he did physiotherapy and swimming.

He underwent the first physical and physiotherapeutic evaluation at our facility in 2019, when he presented with multidistrict hypermobility, valgus knees, a pronated right foot, and dorsal scoliosis. The posterior lower limb muscles were shortened. His walking was uncoordinated, with feet wide apart, and he tended toward a toe-heel gait with slight knee flexion, anterior trunk flexion, and head antepulsion. CMTNS level was 8 (mild gravity), and his level of functioning in daily activities was high (OHS 1). He wore foot orthoses and orthopedic shoes. The strength of the inferior limbs and hands’ pinches is reported in Table 1.

## 3. Physiotherapy and Exercise

### 3.1. Assessment and Exercise Prescription

The physiotherapy program proposed by the medical team from 2019 to 2022 was based on the current state of the literature [10,15,18,19] and tailored to the patient. It included exercises to improve scoliosis and posture; stretching the Achilles tendon and the posterior muscular kinetic chain of the inferior limbs; strengthening the pelvic girdle and proximal inferior limb muscles; improving balance and endurance; and refining gait patterns. To improve his muscle strength, the boy trained in a gym, and ST was operated in a supervised and progressive manner. In an initial phase, care was taken to familiarize the subject with the movements, rarely free weight but with machines, such as Lat Machine, Leg Press, and Shoulder Press; the weight used was adjusted calculating the 1 Repetition Maximum (1 RM) with the formula = w × (36/37 − r), where the w = weight (kg); r = number of repetitions [20]; the repetition range was between 8 and 12, with recovery times between 1 and 2 min; the Time Under Tension (TUT) was free, but such as to allow controlled and fluid movement; all exercises were performed in buffer, i.e., the last repetition of the last set had to be performed without an excessive feeling of stress; a continuous overload was carried out during the program, to guarantee a stimulus that triggered the supercompensation (that means a condition that first decrease physical performance, but with a correct recovery time, become a positive adaptation). In the subsequent phases, four days of training, setting two training days, namely A and B (with different exercise per muscular group trained) repeated twice a week (A, B, A, B); trying to act more on the global load (set per muscular group, repetition, % of 1 RM) of the week than on that of the single training session in order to guarantee a training stimulus which evoke an improvement but which does not burden the condition of CMT.

### 3.2. Periodical Evaluation

The annual evaluation consisted of administering the tests listed below, which are part of the evaluation protocol used for patients with Charcot–Marie–Tooth in our specialized facility [21]. There is no homogeneous consensus about which outcome scales need to be performed in patients with CMT, but in 2009, a meeting including a functional orthopedic surgeon, physiatrist, physiotherapist, and CMT patients organized in our facility selected some functional scales to administer to this kind of patient, helping to understand the patients’ clinical condition before and after rehabilitation treatment. Presently, scientific literature has approved some of these tests for CMT patients. The Medical Research Council (MRC) Scale is used to evaluate the strength of the inferior limbs and hand pinches, commonly graded from 5 (normal) to 0 (no visible contraction). It was initially developed by the Medical Research Council of Great Britain in 1942 to evaluate strength in patients wounded in war or polio. Then, it was described by Kleyweg et al. [22] for use in the peripheral nervous system and requires bilateral examination of the listed muscles. + or − are added if the strength is more or less than the given score. Also, if the scale’s protocol has been well described [23], manual administration makes the score difficult to interpret objectively; therefore, it is suggested that re-evaluation be conducted by the same professional. The Berg Balance Scale is a 14-item objective test used to evaluate balance ability and detect balance impairment and fall risk in people affected by neuromuscular disorders. It can help professionals recognize changes in postural stability, with scores ranging from 0 to 56, where: 0, the patient cannot stand alone; 0–44, there is a risk of fall; 56, there is optimal stability [24]. The Walking Handicap Scale (WHS) is an evaluation tool that assesses walking handicap and disability in domestic and social environments, using a six-category scale ranging from 1 to 6. It is administered by asking about the patient’s walking abilities [25]. The Oxford Handicap Scale (OHS) is based on the Rankin scale used to grade patient disability and participation following a stroke, ranging from 0 (no symptoms) to 5 (total dependence requiring constant attention). It allows monitoring of a patient’s condition over time by scoring their level of independence and restrictions in daily living activities [26]. The 10-Meter Walking Test (10-MHT) [27] is a validated CMT test used to measure physical performance, functional mobility, and walking speed (meters per second) over a short distance. The Manual Ability Measure (MAM-36) is a 36-item self-report questionnaire specific to hand function and handling abilities, validated for patients with CMT [28]. WALK-12 is a test used to assess gait difficulties [29]. Moreover, the CMT Neuropathy Score (CMTNS) was previously graded by a neurologist. CMTNS is a tool composite of nine assessments: 5 of impairment (“sensory symptoms,” “pin sensibility,” “vibration,” “strength arms,” and “strength legs”), 2 of activity limitations (“motor symptoms arms” and “motor symptoms legs”), and two electrophysiological measures (“ulnar CMAP” and “ulnar SNAP”). It is tailored to measure length-dependent motor and sensory impairment in genetic neuropathies. Each assessment is scored on a scale of 0–4 points, reflecting the severity of impairment. Patients are classified as mild (CMTNS ≤ 10), moderate (CMTNS 11–20), or severe (CMTNS > 20) [30]. Based on annual assessments, the subject maintained strength in the distal muscles of the inferior limbs and improved in the proximal regions, particularly the Gluteus Maximus, Gluteus Medius, Quadriceps Femoris, Iliopsoas, and Hamstrings. Pain, Cramps, and Fatigue decreased from 2019 to 2022; postural stability remained nearly the same over the years. Perceived difficulties with walking diminished from 2019 to 2022. Walking speed and disability related to CMT remained nearly the same for years. Clinical examination, posture, and gait have improved over the last two years, while muscle contractures and foot structures have remained unchanged (Table 2).

## 4. Nutritional Intervention and Body Composition Evaluation

### 4.1. Nutritional Intervention

Nutritional diary reported (collected during seven days) a caloric intake of 1600 Kcal, of which around 50 g of protein, therefore less than 1 g × kg of bodyweight, if the goal is increase muscle mass the protein intake, even form vegetable sources, should be at least 1.2 g × kg of body [31]; the initial scheme is shown in the Table 3; vitamins particularly B12 and D was close to the minimum value suggested for sport purpose; even there is no suggested value for polyphenols intake a possible intake to warrant an anti-inflammatory effect should be around 100–200 mg per day; suggested amount per fiber intake is 10 g per 1000 Kcal assumed, so even this nutrient was lacking, suggesting frequent glycemic peaks (the primary regulator of glucose uptake is the amount of fibers) and a possible consequent inflammatory stimulus via Advanced Glycation End-Products (AGEs); our intervention aimed at a slight increase in Kcal, 1800 showed in Table 3.

Therefore, 10% more; an increase in protein intake following the guidelines to promote an increase in muscle mass; an increase in fruit and vegetable consumption to have more polyphenols and fiber; the indications were also to consume a portion of vegetables at the beginning of main meals (lunch and dinner at least) to regulate glycemic spikes; the intake of any food has not been prohibited. We often see the advice to avoid some foods, such as gluten or lactose, because they are considered inflammatory, but there is no supporting scientific literature [32]. The supplements used were whey protein powder, cocoa-flavored (often to enrich breakfast meals), vitamin D (2000 IU), as we noticed it was lacking, and omega-3 fish oil (1 g of DHA + EPA) due to the proven anti-inflammatory action, particularly useful in sports practice [17]; vitamin B12 was supplemented for the first 2 months.

### 4.2. Bioimpedance Analysis

Bioimpedance analysis (BIA) was performed with a bioimpedance analyzer (BIA Anniversary, Akern, Florence, Italy) as follows: after cleansing the skin with alcohol, two electrodes were placed on the right hand and two on the right foot.

BIA analysis was performed in the supine position (i.e., legs positioned at 45° relative to the body’s median line and arms abducted at 30° from the trunk) and before any physical fitness tests. Resistance (R) and reactance (Xc) are the parameters read by the device. The phase angle (PhA) was calculated as [arctan(Xc/R) × 180°/*n*]; the LMI (Levi Mass Index) was calculated as [33,34]; these last two values are particularly sensitive to muscle condition and are more reliable because they are not derived values.

### 4.3. Body Fat Analysis

Body fat analysis was performed using a tissue-scanning technique with the BodyMetrix instrument (Intelametrix, Brentwood, CA, USA), combined with analysis software developed by Hosand Technologies (Verbania, Italy). The instrument is equipped with a 2.5 MHz A-mode ultrasound probe and is connected via USB to a personal computer or tablet.

The software encodes the peaks of the point-by-point return signal in grayscale or blue and, by placing the points next to each other, generates a tissue map of a body region, called stratigraphy. The formula used is Jackson and Pollock with seven points [35,36].

The subject gained 7 kg, almost entirely in muscle mass. The data are corroborated by bioimpedance, stratigraphy, and an overall improvement in physical state. The estimated fat percentage remains practically unchanged (11 to 11.1%), and Phase Angle and LMI improved by +16% and +29%, respectively.

## 5. Discussion

### 5.1. CMT and Physical Exercise: Literature vs. Case Report

To date, rehabilitation is the main effective therapy for people affected by CMT, even if in the scientific literature an established protocol regarding intensity and frequency of physical therapy is still missing [37]. There is strong evidence that resistance training of proximal and distal inferior limb muscles promotes stabilization and improvement of muscle strength in adult and young people with CMT [38]; proprioceptive exercises are recommended to maintain and ameliorate postural balance, and aerobic training is useful to enhance functional ability, aerobic capacity, strength, and fatigue in patients affected by CMT. The use of orthotics for the treatment of this disease is widely discussed but seems to improve balance, dorsiflexion, walking, and fatigue in CMT [39]. Functional surgery is used to restore joint contractions or muscle imbalance, and several articles report that it improves walking pattern, fatigue, cramps, and postural balance in CMT [40]. In summary, progressive and individualized training and physiotherapy programs are recommended and suggested for the treatment of people affected by this disease; moreover, they must be re-evaluated by a multidisciplinary team to update aims and intensity of rehabilitation to maintain their level of functioning and quality of life. This may also be the reason for a lack of standardized treatment protocol for patients with CMT. The physiotherapeutic program used in our report is in line with suggested protocols usually applied; the pivotal difference was in introducing a regular and supervised strength training program. The data collected in our study are consistent with findings in the current literature, demonstrating that complex rehabilitative treatment improves strength, postural stability, fatigue, pain, and cramps in CMT [41]. In particular, in the present case report, the boy focused on strength training and followed a tailored and supervised program in a gym. In current scientific literature, many articles discuss resistance training applied during physiotherapy sessions (typical exercises included, for example, calf raises, bridges, squats without resistance, and use of elastic bands) [10,11,12,13,14,15,19,21], but no one reports results from gym-supervised training in young people. In the report by Djordjevic et al., good results are shown in self-selected exercise, but under constant supervision. Our results are globally in line with articles that reported positive consequences in people who follow resistance training alone or in a supervised group in the gym.

### 5.2. Body Composition and General Evaluation

The subject gained 7 kg, almost entirely in muscle mass. The data are corroborated by bioimpedance measurements, showing improvements in PhA and LMI of +16% and +29%, respectively, confirming an overall increase in muscle mass and physical function. The estimated fat percentage by stratigraphy remains practically unchanged (11 to 11.1%). The subject reports greater self-confidence and a more significant “fit” appearance. The present case report, for the first time in the literature, results from an integrated physical and nutritional plan administered to a boy with CMT1A and followed up for 4 years by the same medical team. In particular, resistance training integrated with diet seemed not only to have no side effects in CMT conditions, at least in the 1a variant considered, but also to be beneficial for managing the pathology. The boy trained productively, which is a result that should also be considered beneficial from a psychological perspective; being able to train and achieve results similar to those of his peers is very important for an 18-year-old [11,21,42]. Probably, the aesthetic motivation helped with compliance with the nutritional guidelines, which undoubtedly played a role in obtaining this good result. The improvement in some muscle areas in terms of strength has undoubtedly contributed to the amelioration of walking, cramps, fatigue, and pain, to improved posture, and, in general, to the subject’s quality of life. Moreover, the obtained results facilitate the boy’s participation in social and recreational activities with his peers. It must be considered that CMT does not have a pharmacological treatment; therefore, achieving symptom stasis or even an improvement is an excellent result.

## 6. Conclusions

Together with a tailored nutritional plan, Training with resistance opposition, if performed in a progressive and mindful manner, and aerobic activity are strongly recommended for children and individuals affected by CMT. Based on the current literature, our experience demonstrates that this kind of multidisciplinary approach can maintain and improve muscle strength, enhance gait pattern, posture, and balance, reduce fatigue, pain, and cramps, and ameliorate mood in patients with CMT. It is undoubtedly a favorable condition that the boy was young and motivated. This favorable situation led to an excellent outcome and can also provide guidance for other CMT patients. Nutritional support should be recommended to support a lifestyle that provides adequate nutrition, especially given the physical activity performed, the subject’s age, and the phenotype. Physiotherapy plays a fundamental role in ensuring regular follow-ups, monitoring the subject’s overall condition, and, if necessary, proposing and modifying a tailored rehabilitation program. It would be interesting to evaluate the inflammatory state, even if it is not easy to implement since these are sub-clinical conditions, therefore the blood values of ESR and PCR are in normal range; on the other hand it is very likely that they are there, therefore we think it is better not to have stimuli that evoke a significant release of inflammatory cytokines, even at the cost of having a minor stimulus in muscle conditioning; in any case, lacking a background literature on ST in CMT, we used an approach that could be absolutely safe for the subject in question and the training sessions were always carried out under the supervision of a sports scientist. Early treatment of CMT allows the patient to be educated on pathology management and have a better quality of life. Moreover, patients’ knowledge and awareness (especially among young people) about a correct lifestyle that includes physical activity and a nutritional plan are still lacking, together with medical professionals’ poor confidence in using this kind of approach in degenerative pathologies like CMT. Currently, there are no standardized protocols; Chetlin et al. [43] reported an improvement in fat free mass and general quality of living with a workout with increasing repetition during time; Jones et al. [44] reported a review with different protocols, some with appreciable results, but with un-homogeneous training programs; same conclusion was drawn by Pedersen et al. [10] that pointed out the possible positive effect of strength training on managing neuropathies but concluded their systematic review highlighting the need for more appropriates studies. In this sense, it is important to consider large-scale studies to provide basic recommendations; however, these must always be personalized given phenotypic variability, even with the same genetic variant, such as CMT1A.

## Figures and Tables

**Table 1:** Medical Research Council scale during the period; units are adimensional and related to the MSC scale.

Muscle	Left				Right			
	2019	2020	2021	2022	2019	2020	2021	2022
Tibial Anterior	4	4	4	4	4	4	4	5
Peroneus	5	4	4	4	5	4	4	5
Extensor Loungus Hallucis	4	3	3	2	4	1	3	2
Triceps Surae	4	3	3	4	4	3	3	4
Flexor Longus Hallucis	4	4	4	4	4	3	4	4
Extensor Communis Ditae	4	4	4	4	4	4	4	4
Gluteus Maximus	4	5	5	5	4	5	5	5
Gluteus Medium	3	3	3	5	2	3	3	5
Quadriceps Femoris	5	5	4	5	5	5	4	5
Iliopsoas	4	5	5	5	4	5	5	5
Hamstrings	4	5	5	5	4	5	5	5
Rectus Abdominis	5	5	5	5	5	5	5	5
Thumb Index Pinch	4	5	5	5	4	5	5	5
Tripod Pinch	5	5	5	5	5	5	5	5
Key pinch	5	5	5	5	5	5	5	5
Hand Grasp	4	5	5	5	4	5	5	5

**Table 2:** Questionnaires were tested during periods.

Test	Years	Years	Years	Years	Percentage (%)
	2019	2020	2021	2022	
VRS PAIN	0	0	0	0	unch
VRS CRAMPS	0	2	0	0	unch
VRS FATIGUE	7	4	0	0	−100
BERG scale	52	54	52	52	unch
WALK-12	35	24	16	13	−62
10 MWT	12	12	11	12	unch
WHS	6	6	6	6	unch
OHS	1	2	1	1	unch
CMTNS	8	7	7	7	unch

VRS: Verbal Rating Scale; 10-MWT: 10 Meters Walking Test; WHS: Walking Handicap Scale; OHS: Oxford Handicap Scale; CMTNS: CMT Neuropathy Score; unch: unchanged.

**Table 3:** Mean weekly dietary intake before and after our intervention.

DIETARY INTAKE	DIETARY INTAKE	DIETARY INTAKE
	BEFORE	AFTER
Energy (Kcal)	1615 ± 110	1825 ± 90
Protein (g)	49 ± 6	85 ± 9
Protein (%TE)	12 ± 2	18.5 ± 1
Carbohydrate (g)	229 ± 10	236 ± 20
Carbohydrate (%TE)	57 ± 8	5 1.8 ±2
Fat (g)	56 ± 10	60 ± 10
Fat (%TE)	31 ± 6	29.6 ± 2
Saturated fat (g)	5 ± 1	5 ± 4
Monounsaturated fat (g)	40 ± 2	50 ± 2
Polyunsaturated fat (g)	5 ± 3	5 ± 2
Cholesterol (mg)	200 ± 20	175 ±15
Fiber (g)	10 ± 4	22 ± 8
Calcium (mg)	1282 ± 357	1270 ± 235
Iron (mg)	12 ± 6	18 ± 5
Magnesium (mg)	342 ± 90	565 ± 54
Zinc (mg)	9 ± 1	11 ± 2
Sodium (mg)	1555 ± 202	1345 ± 242
Potassium (mg)	1899 ± 278	2882 ± 290
Phosphorus (mg)	1310 ± 221	1603 ± 312
Iodine (μg)	139 ± 12	169 ± 18
Thiamin (mg)	2.1 ± 0.5	2.2 ± 0.4
Riboflavin (mg)	3.3 ± 0.7	4.0 ± 1.0
Niacin (mg)	30 ± 1	33 ± 2
Vitamin C (mg)	110 ± 25	300 ± 19
Vitamin A (μg)	800 ± 243	1020 ± 134
Vitamin D (μg)	1 ± 0.7	1.4± 0.2
Vitamin E (mg)	10 ± 1	29 ± 1
Vitamin B6 (mg)	5.3 ± 0.6	6.2 ± 1.5
Vitamin B12 (μg)	1 ± 0.4	2 ± 0.3
Folic acid (μg)	422 ± 52	563 ± 35
Polyphenols (mg)	55 ± 7	180 ± 45

%TE = Percentage of Total Energy Intake. Note: Based on 7-day food records, analyzed and supervised by a registered dietitian nutritionist.

## Data Availability

The data presented in this study are available on request from the corresponding author on request. The data are not publicly available due to privacy and ethical restrictions. Only anonymized data may be shared for scientific research purposes in accordance with applicable ethical and data protection regulations.
